# First metagenome-assembled genome of *Cryptosporidium serpentis* from *Drymarchon couperi* gastric lavage

**DOI:** 10.1128/mra.00342-25

**Published:** 2025-07-31

**Authors:** Mark N. Yacoub, Janina A. Krumbeck, James E. Bogan, Alexandra K. Mason

**Affiliations:** 1 MiDOG Animal Diagnostics LLC 58267, Irvine, California, USA; 2 Central Florida Zoo’s Orianne Center for Indigo Conservation, Eustis, Florida, USA; Rochester Institute of Technology, Rochester, New York, USA

**Keywords:** *Cryptosporidium serpentis*, metagenome, gastric lavage, *Drymarchon couperi*

## Abstract

*Cryptosporidium serpentis* is a protozoan parasite responsible for chronic clinical infections in reptiles, particularly snakes, leading to anorexia and lethargy. Here, we report the first metagenome-assembled genome of *C. serpentis* derived from a gastric lavage sample from the eastern indigo snake, *Drymarchon couperi*.

## ANNOUNCEMENT

*Cryptosporidium* is a protozoan parasite that infects a wide range of animal species. The genomes of 12 *Cryptosporidium* species have been sequenced from mammalian hosts. These species have been implicated as causative agents of intestinal disease including *C. hominis*, *C. muris*, and *C. parvum*. Another species, *C. serpentis*, has been described as a major parasite of snakes. Unlike other *Cryptosporidium* species, which are usually self-limiting, *C. serpentis* remains chronic and frequently causes mortality in infected snakes (1). Despite the pathogen’s significance in reptiles, no genomes of *C. serpentis* have been made publicly available before 2025. Here, we report the first metagenome-assembled genome (MAG) of *C. serpentis*, assembled from a gastric lavage sample from the eastern indigo snake, *Drymarchon couperi*. The genomic data will support accurate identification of *C. serpentis* from metagenomic data and future comparative genomic analysis between *Cryptosporidium* species.

An adult female eastern indigo snake was part of a *C. serpentis* comparative study (2) and tested positive for gastric cryptosporidiosis on histologic examination of a gastric mucosal biopsy, as described by Brownstein et al. (3). *C. serpentis* was identified in gastric lavage samples using a species-specific probe hybridization quantitative Polymerase Chain Reaction (qPCR) assay, which targets the 18S rRNA gene (4). At the time of diagnosis, this snake had not been exhibiting clinical signs typically attributed to gastric cryptosporidiosis. One aliquot of the gastric lavage sample was frozen at −20°C, stored, and shipped overnight to the laboratory for analysis after 2 years.

DNA was extracted from the sample using the ZymoBiomics DNA Miniprep kit and libraries were prepared with the Illumina DNA Prep Kit (Illumina, San Diego, CA) following the manufacturer’s protocol with 10 bp unique dual indexes. The libraries were quantified with Qubit (Thermo Fisher Scientific) and pooled together by equal abundance. The final pool was quantified using qPCR and sequenced on the Illumina Novaseq X, resulting in 174,607,253 reads with 151 bp length, for a total read length of 39,876,739,482 bp. Default parameters were used for bioinformatic tools unless otherwise stated. Read quality was assessed with FastQC v.0.11.8 (5) and reads were trimmed with TrimGalore v.0.5.0 (6). Host sequences were removed by mapping the reads to *Drymarchon couperi* (GCA_043091225.1) with BWA v.0.7.18 (7). The resulting unmapped reads were filtered with Kraken2 v.2.1.3 (8) against the EuPathDB46 database (http://ccb.jhu.edu/data/eupathDB) to remove bacterial sequences, resulting in the identification of 976,347 reads belonging to the *Cryptosporidium* lineage (Sar; Alveolata; Apicomplexa; Conoidasida; Coccidia; Eucoccidiorida; Eimeriorina; Cryptosporidiidae; *Cryptosporidium*). *De novo* genome assembly was performed with MetaSpades v.4.0.0 (9) and the metagenome was binned with MetaBat2 v.2.12.1, producing two bins (bin1 and unbinned) (10). Contigs shorter than 500 bp were removed using BBTools v.39.13 (11). The *C. serpentis* MAG was screened for contamination using the NCBI Foreign Contamination Screen tool (12) with the *C. serpentis* taxid (83999). Genome completeness was assessed using BUSCO v.5.8.2 (13) in genome mode against apicomplexa_odb10 and assembly statistics were collected using QUAST v.5.3.0 (14). Contigs containing telomeres were identified using find_telomeres.py. The resulting genome statistics are presented in Table 1.

**TABLE 1:** Genome assembly statistics for the *C. serpentis* MAG presented in this announcement

Statistic	Value
Number of contigs	4,431
Total genome size (Mbp)	6.67
Coverage (x)	20.53
N_50_	1,833
Size of largest contig (Kbp)	10.7
BUSCO completeness (genome)	84.8
Contamination (bp)	0
Overall GC content (%)	29.80
Telomeres (forward)	13
Telomeres (reverse)	12
Contigs with forward and reverse telomeres	0

## Data Availability

This Whole Genome Shotgun sequence data is available at NCBI under the BioProject PRJNA1234553. The Biosample is available under the accession SAMN47301767. The shotgun sequence reads are deposited at the NCBI Sequence Read Archive (SRA) under the accession SRR32652273. The genomic assembly is deposited under the accession JBMHIZ000000000. Ethical statements and additional sample collection information for the gastric lavage sample was previously published by Bogan et al. [2]. This study was approved by the Central Florida Zoo & Botanical Gardens Research Committee (Project 2023-03).
